# *Escherichia coli* of Ready-to-Eat (RTE) Meats Origin Showed Resistance to Antibiotics Used by Farmers

**DOI:** 10.3390/antibiotics9120869

**Published:** 2020-12-04

**Authors:** Abdulai Abass, Frederick Adzitey, Nurul Huda

**Affiliations:** 1 Department of Animal Science, University for Development Studies, P.O. Box TL 1882, Tamale 1350, Ghana; abdulaiabass.aa@gmail.com; 2 Department of Food Science, University for Development Studies, P.O. Box TL 1882, Tamale 1350, Ghana; 3 Faculty of Food Science and Nutrition, Universiti Malaysia Sabah, Jalan UMS, Kota Kinabalu 88400, Sabah, Malaysia

**Keywords:** antibiotics, *E. coli*, farmers, Ghana, ready-to-eat meats

## Abstract

Bacterial foodborne infections, including meat-derived infections, are globally associated with diseases and some deaths. Antibiotics are sometimes used to treat bacterial infections. The use of antibiotics by farmers contributes to the development of resistance by foodborne pathogens. This study aimed to investigate the antibiotics used by farmers and the occurrence of antibiotic-resistant *Escherichia coli* in ready-to-eat (RTE) meat sources. Data was obtained from livestock farmers through the administration of semistructured questionnaires (*n* = 376) to obtain information on their demographics, knowledge and antibiotic usage. The procedure in the USA Food and Drug Administration (FDA)’s Bacteriological Analytical Manual was used for *E. coli* detection. Antibiotic resistance test was performed using the disk diffusion method. The findings revealed that most of the farmers were male (74.5%), were aged 30−39 years (28.5%), had tertiary education (30.3%) and had 6−10 years of experience in livestock husbandry. Sheep (65.7%) were the most reared livestock, and antibiotics were mostly used to treat sick animals (36.7%). Tetracycline (27.7%) was the most common antibiotic used by farmers, followed by amoxicillin/clavulanic acid (18.6%) and trimethoprim/sulfamethoxazole (11.7%). Most farmers (56.1%) said they had knowledge of antibiotic usage. The prevalence of *E. coli* in RTE meats was lowest in pork (6.0%) and highest in chevon (20.0%). *E. coli* isolates from RTE meats were highly resistant to teicoplanin (96.77%), tetracycline (93.55%), amoxicillin/clavulanic (70.97%), azithromycin (70.97%) and trimethoprim/sulfamethoxazole (58.06%) but was susceptible to chloramphenicol (93.55%), ciprofloxacin (61.29%) and ceftriaxone (58.06%). The multiple antibiotic index ranged from 0.22 to 0.78. Multidrug resistance (93.55%) was high among the *E. coli* isolates. The resistance pattern AmcAzmTecTeSxt (amoxicillin/clavulanic acid–azithromycin–telcoplanin–tetracycline–trimethoprim/sulfamethoxazole) was the most common. The use of antibiotics by farmers must be well regulated. Sellers of RTE meats also ought to take hygiene practices seriously to keep meat safe and healthy for public consumption.

## 1. Introduction

Animal food products, such as egg, meat and milk, are abundant in proteins that are essential for the maintenance, repair and growth of the body. Meat is a food rich in nutrients, containing more bioavailable proteins, vitamins and minerals than other food sources [1]. However, most meats have a high water content that corresponds to about 0.99% water activity, which is suitable for microbial growth [2]. Poultry and red meats are among the most commonly reported carriers of foodborne pathogens [3]. Hughes et al. [4] also asserted that poultry meat, red meat and eggs are recognized as major vectors for transmission of pathogens, such as *E. coli*.

Microorganisms of animal, environmental and human sources normally contaminate raw meat [5,6]. The initial number of microorganisms that may live, including pathogenic or sublethally injured ones, will be substantially reduced when cooking is adequate [7]. However, the prevalence of pathogenic and spoilage microorganisms in ready-to-eat (RTE) meats can be substantially increased by postcooking handling activities, exposure duration at points of sale and meat storage conditions [8]. The common pathogenic bacteria found in RTE meats are *Clostridium perfringens*, *Salmonella enterica, Staphylococcus aureus* and *E. coli* [9,10,11,12]. *E. coli* is the most common facultative anaerobic species found in the gastrointestinal tract of both animals and humans and the most common pathogen present in the family of Enterobacteriaceae [13]. Some types of *E. coli* are responsible for causing diseases by producing Shiga toxins, and they have been linked to several illnesses and some deaths every year [14,15].

RTE meats are popular in Ghana. They are normally sold on the streets, especially at nights, and make a significant contribution to the protein intake of Ghanaians [16]. Meats and foods sold on the streets are generally exposed to foodborne pathogens, including *E. coli*, and are possible sources of foodborne illnesses. Studies carried out in different parts of Ghana have indicated that RTE foods are contaminated with *E. coli*. For instance, *E. coli* was reported in RTE foods in Accra by Agbodaze et al. [17], in Tamale by Abakari et al. [18], in Sunyani by Ofosu et al. [19] and in Bolgatanga by Adzitey et al. [20].

Antibiotics are medications of natural or synthetic origin that have the ability to destroy or prevent growth of microorganisms [21]. They are popularly used for the control, prevention and treatment of infections/diseases [22]. Food animal abuse has important public health consequences as it facilitates the development of bacteria resistant to antibiotics and resistance genes that can be passed on to humans [23]. Abuse happens when farmers use antibiotics intentionally or unintentionally without the requisite knowledge. In addition, the gastrointestinal tracts of farm animals are reservoirs for microorganisms. These microorganisms potentially develop resistance to antibiotics when they are used on the animals [24]. Consequently, transmission to humans might occur when undercooked meat from such farm animals are consumed. Antibiotic resistance in humans can also originate from the environment, pets and wild animals. Microorganisms that are resistant to multiple antibiotics are sometimes widely spread in the environment, pets and wild animals. The environment, pets and wild animals serve as reservoirs and reintroduce resistant bacteria into the food chain, which reach humans via the oral–fecal route or during handling [25].

There is limited study on the knowledge of farmers regarding antibiotic usage, incidence of foodborne pathogens in RTE meats and the antibiotic resistance of foodborne pathogens from RTE meats in Ghana. Therefore, this study aimed to find the antibiotics used by farmers, their knowledge regarding antibiotic usage and prevalence of resistant *E. coli* from RTE meats in Bolgatanga, Ghana.

## 2. Results

### 2.1. Demographic Characteristics and Livestock Species Reared by Farmers

The demographic characteristics and livestock species reared by farmers is shown in Table 1. The majority of farmers were male (74.5%) with age ranging 30−60 years (78.2%). Most of them had either tertiary (30.3%) or nonformal (23.7%) education and had been involved in animal production for 6−10 years (31.6%) or 3−5 years (26.9%). The farmers reared sheep (*n* = 247), chicken (*n* = 246), goats (*n* = 243), cattle (*n* = 177), guinea fowls (*n* = 175) and pigs (*n* = 93).

### 2.2. Usage of Antibiotics by Livestock Farmers

**Figure 1:**
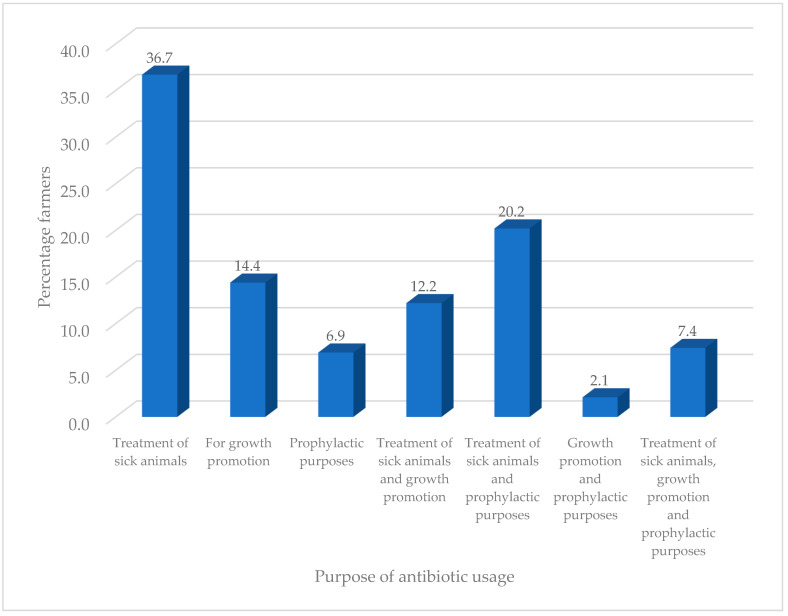
Purpose of antibiotic usage by farmers.

**Figure 2:**
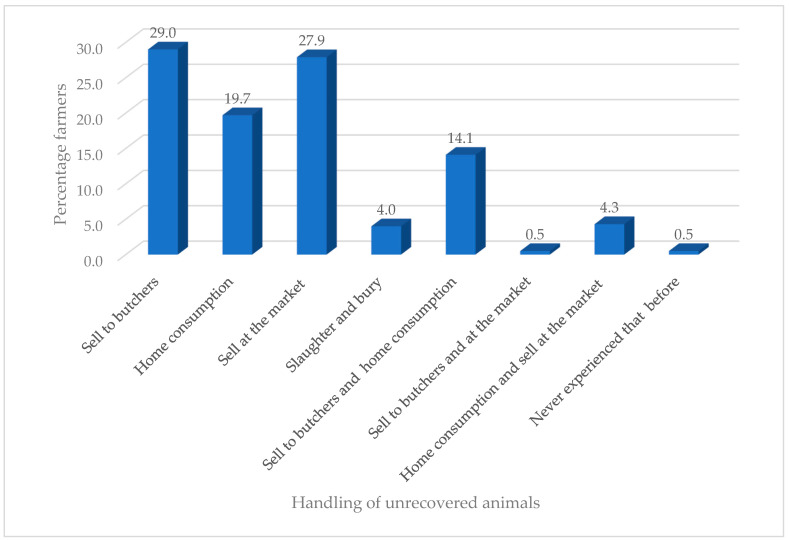
Handling of unrecovered animals.

The majority of farmers (92.8%) had experienced infections on their farm and mostly consulted veterinary officers (77.7%) for advice (Table 2). The farmers mostly used tetracycline (27.7%) to treat their animal, while the least used antibiotics were penicillin (4.3%) and erythromycin (4.3%). Recommendations for antibiotic usage mostly came from colleague farmers (35.1%) and veterinarians (31.6%) and the least from veterinarians and drug sellers (2.1%). The majority of farmers said they had knowledge of antibiotic usage (56.1%), and they got this from colleague farmers (32.7%) and veterinary officers (21.8%). The educational level of farmers had no influence on their knowledge of antibiotic usage (*χ*^2^ = 5.651a, df = 10, *p* = 0.844) but had influence (*χ*^2^ = 32.158a, df = 5, *p* = 0.000) on the type of antibiotics used. The years of experience in livestock production had no influence on their knowledge of antibiotic usage (*χ*^2^ = 8.460a, df = 8, *p* = 0.390) and on the type of antibiotics used (*χ*^2^ = 5.701a, df = 4, *p* = 0.223). Many farmers (67.6%) were involved in treating their animals but observed safety dosage instructions (72.1%) and withdrawal periods (65.7%). Antibiotics were used purposely for treatment of sick animals (36.7%), and only few used it for prophylactic and growth purposes (2.1%) (Figure 1). The majority of farmers sold their unrecovered animals after treatment to butchers (29.0%), whilst those who had never experienced that situation (0.5%) were the least common (Figure 2).

### 2.3. Prevalence of E. coli in Ready-to-Eat Meats

**Figure 3:**
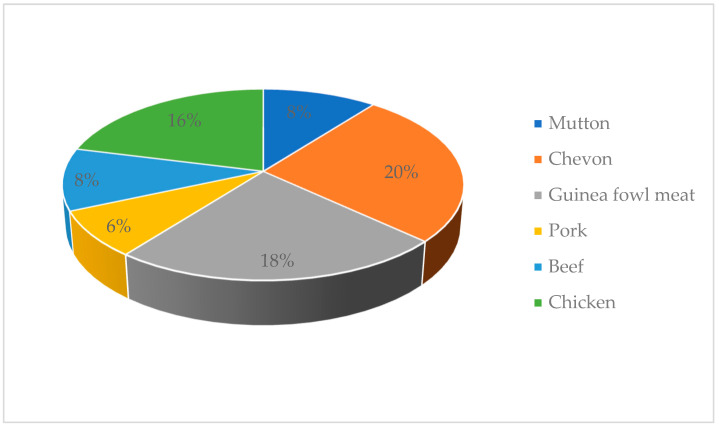
Prevalence of *E. coli* in ready-to-eat meats.

The prevalence of *E. coli* in RTE meats is presented in Figure 3. Ten samples of RTE chevon (20%), nine samples of guinea fowl (18%), eight samples of chicken (16%), four samples of beef (8%), four samples of mutton (8%) and three samples of pork (6%) were contaminated by *E. coli*.

### 2.4. Antibiotic Resistance of E. coli from RTE Meats

The *E. coli* isolates showed high resistance to teicoplanin (96.77%), tetracycline (93.55%), amoxicillin/clavulanic acid (70.97%) and azithromycin (70.97%) (Table 3). Relatively high intermediate resistance was observed for gentamicin (38.71%), sulfamethoxazole/trimethoprim (25%), ceftriaxone (22.58%), azithromycin (16.13%) and ciprofloxacin (16.13%). The *E. coli* isolates were highly susceptible to chloramphenicol (93.55%).

### 2.5. Multidrug Resistance of Individual E. coli Isolates

The antibiotic resistance pattern of the RTE meats can be found in Table 4. The multiple antibiotic (MAR) index ranged from 0.22 to 0.78. The 31 *E. coli* exhibited 24 different resistance profiles. In addition, 6.45%, 16.13%, 22.58%, 32.26%, 12.90% and 9.68% were resistant to two, three, four, five, six and seven antibiotics, respectively.

## 3. Discussions

Resistance of bacteria to antibiotics is essentially linked to their usage either as prophylactic treatment of diseases or as growth promoters [22,23]. This phenomenon is a worldwide issue and threatens public health [23]. Farmers are among the primary users of antibiotics, which ends up in animals, the environment and eventually humans as a result of consumption [26,27]. To ascertain this, we studied livestock farmers’ knowledge and usage of antibiotics and the sensitivity of *E. coli* from RTE meat sources to antibiotics. In our study, males and the youth dominated the livestock rearing business, which could be attributed to the fact that males in Northern Ghana mostly keep animals as their main source of work and for payment of dowry and most often own animals kept by women as the head of the family. Similar findings have been reported by Akansale et al. [28] and Ozturk et al. [29]. The farmers who kept ruminants and fowls made up the majority due to the usefulness of these animals for religious purposes and the ready market availability. Those who reared pigs were few in number due to religious restrictions, particularly Islam, which forbids Muslims from keeping and consuming swine. Education and experience played a key role in the acquisition, acceptance and application of knowledge. A higher proportion of farmers had tertiary education compared to nonformal education and had appreciable years of experience in livestock rearing. This reflected and contributed to their knowledge and usage of antibiotics as the majority consulted veterinarians when they experienced infections on their farm. In addition, the majority said they had knowledge of antibiotic usage and observed withdrawal periods and safety measures and dosage instructions when administering antibiotics. There was no relationship between the level of education and knowledge of antibiotic usage, although this did exist with the type of antibiotics used. There was no relationship between the years of experience and knowledge of antibiotic usage and the type of antibiotics used. Farmers acquired information on antibiotics and their usage from experienced people (veterinary officers, extension officers and colleague farmers) and organizations (nongovernmental organizations (NGOs)). They mainly used the following antibiotics to treat animals: gentamicin, tetracycline, amoxicillin/clavulanic acid, trimethoprim/sulfamethoxazole, ciprofloxacin, erythromycin, chloramphenicol and penicillin. This finding is similar to the work of Adesokan et al. [26], who reported that beta-lactams/aminoglycosides (20.4%), fluoroquinolones (26.5%) and tetracycline (33.6%) formed the majority of antibiotics used by farmers in livestock production in Nigeria. Katakweba et al. [27] also identified tetracycline (61%), sulfadimidine (23%), penicillin–streptomycin (2%) and gentamycin (1%) as the most used and commonly reported antibiotics among livestock keepers in Tanzania. In this study, farmers received recommendations to use a particular antibiotic from relevant people, such as veterinarians, colleague farmers and veterinary drug sellers. This study also found that antibiotics were used mainly for prophylaxis, treatment of sick animals and as growth promoters. These findings agree with that of Ferdous et al. [30], who found that antibiotics were used by farmers for prophylactic purposes (14.17%), therapeutic purposes (34.16%) or for both therapeutic and prophylactic purposes (40.83%). Oluwasile et al. [31] revealed that four (6.9%) poultry farmers used antibiotics as growth promoters. It is bad practice for farmers to sell or consume animals that do not recover from their sickness following antibiotic treatments. These animals may end up at the abattoir and processed into meats, which will enter the food chain. The meats produced from these animals are potential sources of antibiotic-resistant bacteria, which can easily be consumed by humans, thus increasing the antibiotic resistance burden.

The RTE meats in this study were sources of *E. coli*, with the prevalence being highest in chevon and lowest in pork. The presence of *E. coli* in RTE meats is due to poor pre- and postcooking handling practices, resulting in direct or indirect fecal and/or environmental contamination. Pathogenic *E. coli* is known to cause kidney failure, low blood platelets and hemolytic anemia [32]. In Italy, Nobili et al. [33] isolated *E. coli* from RTE hamburger and beef carpaccio, and these *E. coli* strains were positive for *vtx1* genes. Outbreaks of Shiga toxin-producing *E. coli* (STEC) associated with the consumption of RTE cooked and processed meat products in Australia [34] and deli meats in the USA [35] have been reported. This study is also comparable to the findings of Hassanin et al. [36], who reported that *E. coli* were present in RTE meats such as hawawshi (46.7%), kofta (40%) and shawerma (33.3%) in a study conducted in Egypt. Ahmadi et al. [37] examined RTE meats and meat products in India and reported that meat curry (12.12%) and nonveg momo (4.0%) were positive for *E. coli*. In Bangladesh, Rahman et al. [38] reported that RTE chicken (49.02%) and beef (70.00%) samples were contaminated by *E. coli*. *E. coli* was detected in 32.0% of RTE meats from Latvia [39]. Contrary to the present work, Syne et al. [10] did not find *E. coli* in RTE meats sampled in Trinidad but did detect *E. coli* in some precooked RTE samples. In Europe, low contamination rates (2.21%) of STEC were reported in meat products and meat preparations from mixed sources [40].

The RTE *E. coli* isolates exhibited >50% resistance to teicoplanin, amoxicillin/clavulanic acid, tetracycline, azithromycin and sulfamethoxazole/trimethoprim. Lower (19–23%) resistance was observed for ceftriaxone, ciprofloxacin and gentamicin. Among the antibiotics used by livestock farmers in this study to treat their animals were tetracycline, ciprofloxacin, amoxicillin/clavulanic acid and gentamicin. Tetracycline and amoxicillin/clavulanic acid were used by most farmers, and high resistance to these antibiotics were exhibited by RTE *E. coli* isolates. Both tetracycline and amoxicillin/clavulanic acid are broad-spectrum antibiotics. Tetracycline destroys bacteria by inhibiting protein synthesis, while amoxicillin/clavulanic acid does the same by inhibiting biosynthesis of the peptidoglycan layer in the cell wall [41,42]. A relatively higher proportion of *E. coli* species showed intermediate resistances to gentamicin, sulfamethoxazole/trimethoprim, ceftriaxone, ciprofloxacin and azithromycin. Intermediate resistance suggests potential future resistance, and such isolates are cumbersome to handle or must be destroyed when they are responsible for causing infections [43,44,45]. Harakeh et al. [46] reported that *E. coli* of RTE meats origin were 100% resistant to trimethoprim/sulfamethoxazole and teicoplanin and 88.9% resistant to erythromycin, clindamycin, vancomycin and oxacillin. This study also found high resistance to teicoplanin but lower resistance to trimethoprim/sulfamethoxazole. Susceptibility to gentamicin was very low in this study compared to that of Harakeh et al. [46], which was 100%. Rahman et al. [38] reported that *E. coli* from RTE meats were resistant to amoxicillin (76.00%), sulfonamide–trimethoprim, (84.00%) and oxytetracycline (92%), while *E. coli* of RTE from beef showed resistance to oxytetracycline (92%) but susceptibility to gentamicin (100%) and ciprofloxacin (100%). This study found lower susceptibility to gentamicin and ciprofloxacin. Multidrug resistance (resistance to three or more antibiotics) was observed among most of the RTE meat *E. coli* isolates. Most of the isolates also exhibited different resistance profiles and were either resistant to two (6.45%), three (16.13%), four (22.58%), five (32.26%), six (12.90%) or seven (9.68%) antibiotics. Similar findings were made by Harakeh et al. [46] and Rahman et al. [38]. Multidrug resistance has been linked to the use of antibiotics in livestock production for prophylactics, treatment of animals and as growth promoters [10,47], which is in agreement with the findings of this study.

## 4. Materials and Methods

### 4.1. Study Area

The study was carried out in Bolgatanga, Ghana. Bolgatanga is the Upper East regional capital and situated between latitudes 10°30’ and 10°50’ North and longitudes 0°30’ and 1°00’ West [48]. Bolgatanga is urban and has a population of 66,685 [48].

### 4.2. Study Population, Sample Size and Sampling Method

The study population was ruminant and nonruminant farmers who were willing to take part in the study. The sample size was 376 livestock farmers, and they were selected using snow ball sampling technique. According to this, each farmer we contacted referred us to and recruited other farmers willing to take part in the survey. The sample size was obtained by querying the population of livestock farmers in the sample size calculator [49], and a sample size of 376 was obtained at a confidence level of 95%.

### 4.3. Structure of Questionnaire

The study used semistructured questionnaires, which included both open- and close-ended questions to collect information from livestock farmers (the questionnaire used has been provided as Supplementary Materials). The participants’ personal details, the antibiotics used for treatments to improve animal health and farmers’ general knowledge of antibiotics were assessed.

### 4.4. Sampling of Ready-to-Eat Meats

Sampling was conducted from January to October, 2019. Five samples each of mutton, chevon, beef, pork, chicken and guinea fowl were collected each month. In total, 300 RTE meat samples comprising 50 mutton, 50 chevon, 50 beef, 50 pork, 50 chicken and 50 guinea fowl meats were collected randomly from street vendors to investigate the existence of *E. coli*. Sterile surface swab template (10 cm^2^) was placed on the surface of each RTE meat and swabbed with a sterile swab stick. The swabbed samples of RTE meat were transported under 4 °C to the laboratory for microbial analysis.

### 4.5. Isolation and Confirmation of E. coli

This was done using a slightly modified method of Feng et al. [50] and Adzitey et al. [12]. Swabs were broken into universal bottles containing 9 mL of buffered peptone water and incubated for 24 h at 37 °C. A loopful of culture from the buffered peptone water was streaked onto Levines’s eosin methylene blue (LEMB) agar and then incubated for 24 h at 37 °C. Presumptive *E. coli* isolates on the LEMB agar were streaked onto tryptic soy agar and incubated for 24 h at 37 °C for purification. Three to five presumptive *E. coli* isolates from each plate were confirmed by Gram staining, growth in brilliant green bile with Durham tubes, growth in nitrate agar and latex agglutination test for *E. coli*. All media used were purchased from Oxoid Limited, Basingstoke, UK.

### 4.6. Antimicrobial Susceptibility Test

This was done using the disk diffusion method by Bauer et al. [51]. A total of nine antibiotics—amoxicillin/clavulanic acid 30 µg (Amc), azithromycin 15 µg (Azm), ceftriaxone 30 µg (Cro), chloramphenicol 30 µg (C), ciprofloxacin 5 µg (Cip), gentamycin 10 µg (Cn), teicoplanin 30 µg (Tec), tetracycline 30 µg (Te) and sulfamethoxazole/trimethoprim (Sxt)—were used. Purified cultures of *E. coli* were grown for 16−18 h in trypticase soy broth (TSB) at 37 °C, after which the cultures were adjusted to 0.5 McFarland solution and spread on the surface of Mueller–Hinton agar using sterile cotton swabs. Four or five antibiotic disks were placed on the surface of the Mueller–Hinton agar plates at a distance to avoid overlapping of inhibition zones. The plates were incubated overnight at 37 °C. Inhibition zones were measured, and the results were interpreted as sensitive, intermediate resistant or resistant according to Clinical and Laboratory Standards Institute guidelines [52]. All media and antibiotic disks used were purchased from Oxoid Limited, Basingstoke, UK.

### 4.7. Analysis of Data

Results from the questionnaires were analyzed using SPSS (Statistical Package for Social Sciences) version 20. Chi square test (χ^2^) was used to show the relationships between some of the parameters, and significant differences were considered when *p* ≤ 0.05.

## 5. Conclusions

The antibiotics used by farmers have a link with the resistance pattern of bacteria isolated from the animals they use them on, which may end up in meat and meat products. The RTE meats in this study were sources of resistant *E. coli* isolates. Therefore, RTE meats in Ghana is a potential public health hazard and could be a route for transmission of antibiotic-resistant *E. coli*. This study recommends training farmers on antibiotics and their usage and training RTE meat processors on hygienic meat handling.

## Figures and Tables

**Table 1:** The demographic characteristics of livestock farmers.

Variable	Frequency	Percentage
Gender		
Male	280	74.5
Female	96	25.5
Age		
20–29	48	12.8
30–39	107	28.5
40–49	103	27.4
50–60	84	22.3
61 and above	34	9.0
Educational status		
Nonformal	89	23.7
Primary	60	16.0
Junior high	54	14.4
Senior high	50	13.3
Tertiary	114	30.3
Others	9	2.4
Years in livestock business		
0–11 months	24	6.4
1–2 years	61	16.2
3–5 years	101	26.9
6–10 years	119	31.6
More than 10 years	71	18.9
Species of animals reared		
Sheep	247	65.7
Chicken	246	65.4
Goats	243	64.6
Cattle	177	47.1
Guinea fowls	175	46.5
Pigs	93	24.7

**Table 2:** The knowledge and usage of antibiotics by livestock farmers.

Variable	Frequency	Percentage
Have you ever encountered infections on your farm?		
Yes	349	92.8
No	27	7.2
Did you consult a veterinarian?		
Yes	292	77.7
No	84	22.3
Which antibiotics did you use?		
Gentamicin	27	7.2
Tetracycline	104	27.7
Amoxicillin/clavulanic acid	70	18.6
Trimethoprim/sulfamethoxazole	44	11.7
Ciprofloxacin	29	7.7
Erythromycin	16	4.3
Chloramphenicol	37	9.8
Penicillin	16	4.3
Herbal drug	33	8.8
Who recommended the antibiotics?		
Colleague farmers	132	35.1
Veterinarians	119	31.6
Drug sellers	68	18.1
Colleague farmers and veterinarians	14	3.7
Colleague farmers and drug sellers	26	6.9
Drug sellers and veterinarians	8	2.1
Colleague farmers, drug sellers and veterinarians	9	2.4
Would you say you have knowledge of antibiotics usage?		
Yes	211	56.1
No	165	43.9
Where did you get the information from?		
Extension officers	56	14.9
Nongovernmental organization (NGO)	45	12.0
Colleague farmers	123	32.7
Veterinary staff	82	21.8
Extension officers and NGO	10	2.7
Extension officers and colleague farmers	27	7.2
Extension and veterinary staff	8	2.1
NGO and colleague farmers	15	4.0
NGO and veterinary staff	3	0.8
Extension officers, NGO, colleague farmers and veterinary staff	7	1.9
Have you ever treated your animals yourself?		
Yes	254	67.6
No	122	32.4
Did you observe safety and dosage instructions?		
Yes	271	72.1
No	105	27.9
Do you follow antibiotic withdrawal period instructions?		
Yes	247	65.7
No	129	34.3

**Table 3:** Percentage antibiotic resistance of *E. coli* from ready-to-eat meat samples.

Antimicrobial	%R	%I	%S
Amoxicillin/clavulanic acid 30 µg (Amc)	70.97	0.00	29.03
Azithromycin 15 µg (Azm)	70.97	16.13	12.90
Ceftriaxone 30 µg (Cro)	19.35	22.58	58.06
Chloramphenicol 30 µg (C)	6.45	0.00	93.55
Ciprofloxacin 5 µg (Cip)	22.58	16.13	61.29
Gentamicin 10 µg (CN)	22.58	38.71	38.71
Teicoplanin 30 µg (Tec)	96.77	0.00	3.23
Tetracycline 30 µg (Te)	93.55	6.45	0.00
Sulfamethoxazole/trimethoprim (Sxt)	58.06	25.81	16.13

S, susceptible; I, intermediate resistant; R, resistant.

**Table 4:** Antibiotic resistance profile and multiple antibiotic resistance (MAR) index of *E. coli.*

Code	Sources	Number of Antibiotics	Antibiotic Resistance Profile	MAR Index
GO18	Goat	7	CipAmcAzmTecTeCSxt	0.78
P12	Pig	7	AzmTecCnTeCCroSxt	0.78
C17	Chicken meat	7	AmcAzmTecCnTeCroSxt	0.78
GO4	Goat	6	AmcAzmTecTeCroSxt	0.67
P5	Pig	6	CipAmcTecCnTeSxt	0.67
B7	Beef	6	CipAmcAzmTecTeSxt	0.67
C19	Chicken meat	6	AmcAzmTecCnTeSxt	0.67
GO1	Goat	5	CipAmcTecTeCro	0.56
GO16	Goat	5	AmcAzmTecCnTe	0.56
S26	Sheep	5	AzmTecTeCroSxt	0.56
G33	Guinea fowl	5	AmcTecTeCroSxt	0.56
B37	Beef	5	AmcAzmTecTeSxt	0.56
C7	Beef	5	AmcTecCnTeSxt	0.56
C13	Chicken meat	5	AmcAzmTecTeSxt	0.56
C18	Chicken meat	5	AmcAzmTecTeSxt	0.56
C20	Chicken meat	5	AmcAzmTecTeSxt	0.56
C23	Chicken meat	5	CipAmcAzmTecTe	0.56
GO30	Goat	4	AzmTecTeSxt	0.44
GO40	Goat	4	AmcAzmTecTe	0.44
S11	Goat	4	AzmTecTeSxt	0.44
G11	Pig	4	CipAmcAzmTec	0.44
B4	Guinea fowl	4	AmcAzmTecSxt	0.44
B49	Beef	4	CipAmcAzmTe	0.44
C15	Chicken meat	4	AmcAzmTecTe	0.44
G046	Goat	3	AmcAzmSxt	0.33
GO49	Goat	3	AzmTecTe	0.33
P3	Sheep	3	TecTeSxt	0.33
G14	Guinea fowl	3	AzmTecTe	0.33
G30	Guinea fowl	3	TecCnTe	0.33
GO6	Goat	2	TecTe	0.22
S3	Sheep	2	TecTe	0.22

## Supplementary Materials

The questionnaire used for the study is available online at https://www.mdpi.com/2079-6382/9/12/869/s1.
