# Supplementary material for: Escherichia coli of Ready-to-Eat (RTE) Meats Origin Showed Resistance to Antibiotics Used by Farmers
Source: Antibiotics (Basel). 2020 Dec 4;9(12):869. doi: 10.3390/antibiotics9120869 (PMC7761968; doi:10.3390/antibiotics9120869)
Supplement: Supplementary file 1 [file antibiotics-09-00869-s001.pdf]

UNIVERSITY FOR DEVELOMENT STUDEIS
FACULTY OF AGRICULTURE
DEPARTMENT OF ANIMAL SCIENCE

A SURVEY ON ANTIBIOTICS USAGE FOR LIVESTOCK FARMERS

This study is to identify the most frequently used antibiotics, their dose, time of use and the withdrawal times prior to market or slaughter. Please, information given will be treated with high level of confidentiality.
Please fill the questions below as best as you can. Tick where appropriate [√]

A. PERSONAL DATA

1. Gender: a. Male [ ] b. Female [ ]
2. Age group. a. 20-29 [ ] b. 30-39 [ ] c. 40-49 [ ] d. 50-60 [ ] e. 61 & above [ ]
3. Educational level. a. Non formal [ ] b. Primary school [ ] c. Junior high school [ ] d. Senior High school [ ] e. Tertiary [ ] f. Others [ ]
4. How many years have you been in this business? a. 0-11 months [ ] b. 1-2 years [ ] c. Between 3 - 5 years [ ] d. 6-10 years [ ] e. above 10 years [ ]
5. What type of animals do you rear? a. Cattle [ ] b. Goat [ ] c. Sheep [ ] d. Pig [ ] e. Guinea fowl [ ] f. Fowl [ ] e. Others [ ]

B. THE FARMER'S KNOWLEDGE ON ANTIBIOTICS AND THEIR USAGE

6. Have you ever encountered any infection in the animals on your farm? a. Yes [ ] b. No [ ]
7. If yes, did you consult veterinary officers/technicians/animal health officers to know what kind of infection it was? a. Yes [ ] b. No [ ]
8. What type of antibiotic did you use? a. Gentamicin [ ] b. Tetracycline [ ]
   c. Amoxycillin/clavulanic [ ] d. Trimethoprim/sulfamethoxazole [ ] e. Ciprofloxacin [ ] f. Erythromycin [ ] g. Chloramphenicol [ ] h. Others specify…………..
9. Who recommended the antibiotics for you? a. Colleague farmer [ ] b. Veterinarian [ ] c. Drug seller d. Others ………………………………
10. Would you say you have knowledge on the antibiotic usage? a. Yes [ ] b. No [ ]
11. If yes, who/where did you get the information from? a. Extension officers [ ] b. NGOs [ ] c. Colleague farmers [ ] d. Veterinary staff e. Others [ ]
12. For what purpose do you use antibiotics? A. Treat sick animals [ ] b. For growth

promotion [ ] c. Prophylactic purposes [ ] d. Others specify ……………………

13. Who administers the antibiotic to the animals? a. self [ ] b. veterinary officer [ ] c. both. d. Others specify…………………………
14. If self or both do you observe safety and dosage instructions for the antibiotic?
    a. Yes [ ] b. No [ ]
15. Do you follow antibiotic withdrawal period instructions? a. Yes [ ] b. No [ ]
16. In case the treated animal is not recovering, what do you do to the animal? a. sell to butchers [ ] b. home consumption [ ] c. market [ ] d. others specify……………
